# An optimized culture system for notochordal cell expansion with retention of phenotype

**DOI:** 10.1002/jsp2.1028

**Published:** 2018-07-26

**Authors:** Matthew D. Humphreys, Lizzy Ward, Stephen M. Richardson, Judith A. Hoyland

**Affiliations:** ^1^ Division of Cell Matrix Biology and Regenerative Medicine, School of Biological Sciences, Faculty of Biology, Medicine and Health University of Manchester Manchester UK; ^2^ NIHR Manchester Musculoskeletal Biomedical Research Unit, Central Manchester Foundation Trust, Manchester Academic Health Science Centre Manchester UK

**Keywords:** culture systems, degeneration, tissue specific progenitor cells

## Abstract

**Background:**

Notochordal (NC) cells display therapeutic potential in treating degeneration of the intervertebral disc. However, research on their phenotype and function is limited by low‐cell yields and a lack of appropriate methodology for cell expansion. Utilizing porcine cells, this study aimed to develop an optimized culture system which allows expansion of NC cell populations with retention of phenotype.

**Methods:**

Post‐natal porcine and foetal human nucleus pulposus tissue was compared histologically and expression of known NC cell marker genes by porcine NC cells was analyzed. Porcine NC cells were isolated from six‐week post‐natal discs and cultured in vitro under varied conditions: (1) DMEM vs αMEM; (2) laminin‐521, fibronectin, gelatin and uncoated tissue culture‐treated polystyrene (TCP); (3) 2% O_2_ vs normoxia; (4) αMEM (300 mOsm/L) vs αMEM (400 mOsm/L); (5) surface stiffness of 0.5 and 4 kPa and standard TCP. Adherence, proliferation, morphology and expression of NC cell markers were assessed over a 14‐day culture period.

**Results:**

Native porcine nucleus pulposus tissue demonstrated similar morphology to human foetal tissue and porcine NC cells expressed known notochordal markers (CD24, KRT8, KRT18, KRT19, and T). Use of αMEM media and laminin‐521‐coated surfaces showed the greatest cell adherence, proliferation and retention of NC cell morphology and phenotype. Proliferation of NC cell populations was further enhanced in hypoxia (2%) and phenotypic retention was improved on 0.5 kPa culture surfaces.

**Discussion:**

Our model has demonstrated an optimized system in which NC cell populations may be expanded while retaining a notochordal phenotype. Application of this optimized culture system will enable NC cell expansion for detailed phenotypic and functional study, a major advantage over current culture methods described in the literature. Furthermore, the similarities identified between porcine and human NC cells suggest this system will be applicable in human NC cell culture for investigation of their therapeutic potential.

## 1 INTRODUCTION

The increasing incidence of discogenic low‐back pain and inability of current treatments to offer long‐term resolution has necessitated development of alternative therapies. The drive towards cell‐based regenerative therapies for degeneration of the intervertebral disc (DIVD) has resulted in a focus on identification of suitable cell types to drive repair/regeneration. This has included nucleus pulposus (NP) cells, and notochordal (NC) cells, which in humans are only found in the IVD during embryonic/foetal development and throughout childhood. There is compelling evidence to suggest that NC cells are the early progenitors for adult NP cells^1^ and that the disappearance of cells with the recognizable NC‐like morphology (ie, large, rounded and vacuolated cells) and the onset of DIVD are associated chronologically, which may imply causation.^2^, ^3^ As such, NC cells are thought to have substantial therapeutic application and may promote a healthy phenotype in adult NP cells of several species^4^, ^5^, ^6^ and NP tissue explants.^7^

The NP matrix consists of an irregular mesh of type II collagen and elastin fibers with abundant and highly hydrophilic proteoglycans, most significantly aggrecan.^8^, ^9^, ^10^, ^11^, ^12^ DIVD commonly results in a change from type II to type I collagen and a decrease in proteoglycan content leading to dehydration of the NP,^13^ an increase in expression of matrix degrading enzymes,^14^, ^15^, ^16^, ^17^ an upregulation of inflammatory cytokines^18^, ^19^, ^20^ and an increase in cell senescence and apoptosis.^21^, ^22^, ^23^ Adult NP cells cultured in NC cell conditioned media (NCCM) showed an upregulation of aggrecan, type II collagen, CD44, link protein and tissue inhibitor of metalloproteinase 1 (TIMP1).^24^, ^25^, ^26^, ^27^ In addition, NC cell secreted factors protect NP cells from proinflammatory cytokines, for example, IL‐1β, TNF‐α, and IL‐6, matrix metalloproteinases (MMPs) and activated caspases.^24^, ^28^, ^29^, ^30^, ^31^ These regenerative effects are thought to be mediated via a range of potentially therapeutic bioactive factors including connective tissue growth factor (CTGF).^28^, ^29^ Taken together, this evidence provides rationale for the use of NC cells in cell‐based regenerative therapies for DIVD and treatment of the associated low‐back pain.

However, current culture systems for NC cells, such as the gold standard alginate bead culture, do not allow for proliferation of NC cell populations.^5^, ^6^, ^32^, ^33^, ^34^, ^35^, ^36^ Thus even in commonly used animals where NC cells are retained throughout the majority of lifespan, such as porcine,^7^, ^34^, ^37^ canine^24^, ^36^ and rat,^38^, ^39^ multiple disc levels or multiple animals are required to pool sufficient cells for experimentation. This represents a significant limitation both in current studies investigating NC cell phenotype and function, as well as for future potential clinical translation of human cells. Such limitations highlight the urgent need for optimized methodologies to expand populations of NC cells without loss of phenotype.

Monolayer culture of NC cells on tissue culture‐treated polystyrene (TCP) has been shown to promote proliferation,^32^ but has shown poor retention of phenotype, in both porcine and bovine models.^35^, ^40^ As such, optimization of culture conditions and consideration of microenvironmental factors are essential to enhance attachment, proliferation and retention of phenotype. Work from Rastogi et al, indicated that choice of media affected NC cell adherence and proliferation, and expression of NC genes such as CD24 in rat NC cells.^38^ αMEM and DMEM media were shown to be the most preferable for attachment, and αMEM for retention of gene expression. Coating of two‐dimensional (2D) surfaces for monolayer culture has been suggested to promote greater adherence of NC cells, particularly those using laminin isoforms such as 332 and 511.^37^, ^41^ In addition to surface coating, substrate stiffness is also an important consideration. While TCP has a stiffness in the GPa range, the stiffness of a juvenile NP is ~0.3 kPa and mature NP ~5 kPa^37^, ^42^ and soft laminin‐coated substrates have been shown to have a profound effect on immature NP cell morphology and gene expression.^43^ Other microenvironmental factors have also been important in modulating both NP cell and NC cell phenotype and function, including hypoxia,^34^, ^38^, ^44^ osmolarity^36^, ^45^, ^46^ and mechanical loading^47^, ^48^, ^49^, ^50^ and hence these factors may be key when optimizing physiologically‐relevant culture systems for expansion of NC cells with retention of phenotype.

Here, we used porcine NC cells to develop a system that could be used for expansion of NC cell populations with retention of phenotype over a 14‐day culture period. A porcine system was chosen as this system has been shown to contain 80% to 88% NC cells within their NP throughout life and to consistently yield a large number of cells.^37^, ^51^ We define a monolayer culture system which allows proliferation of NC cells with significant retention of NC phenotype by consideration of media choice, surface coating, hypoxia, media osmolarity and surface stiffness.

## 2 METHODS

### 2.1 Human foetal sample processing

A whole human foetal spine (18‐week post‐conception) was obtained with written informed donor consent and National Research Ethics Service Committee approval. Tissue was processed for histological comparison with porcine tissue, with all methodology performed in accordance with the Committee's and Human Tissue Authority (HTA) guidelines. Tissue was incubated overnight in 40 mL of formal saline at room temperature with agitation, then transferred to 40 mL EDTA (20% pH 7.4) and incubated for a further 72 hours. Following this, the sample was washed overnight with running water and processed into paraffin blocks Paraffin sections (5 μm) were then stained with hematoxylin and eosin, Masson's trichrome, alcian blue (pH 2.5)/aqueous neutral red or safranin‐O/fast green according to established protocols. Images were taken using three‐dimensional (3D) Histech Pannoramic 250 Flash Slide Scanner and Pannoramic Viewer.

### 2.2 Porcine sample processing

Porcine carcasses (6‐week‐old, female, 10‐12 kg suckling pigs) were obtained from an abattoir in accordance with local regulations. Spines were removed by dissection under non‐sterile operating theater conditions and transferred to a sterile tissue culture hood where individual discs were dissected out. Discs were then either processed individually for histological staining as described above, or NP and AF tissue isolated for gene expression analysis.

### 2.3 Porcine notochordal cell culture

NP tissue was dissected as above and used for isolation of cells by overnight incubation at 37°C with agitation in 0.0125% collagenase II (Gibco, Fisher Scientific UK Ltd, Loughborough). Cells were then centrifuged and resuspended in 20 mL warm cell dissociation solution (Sigma‐Aldrich, Gillingham, UK) for 15 minutes. Cells were washed and plated at 1 × 10^5^ cells in 1 mL of media per well into six‐well plates or MatTek dishes (35 mm Glass bottom dish, 14 mm micro‐well). Alginate beads (1.2% v/v) were prepared using established protocols at a density of 4 × 10^6^ cells/mL. Following culture in alginate beads, cells were harvested by incubation with dissolving buffer (55 mM Sodium Citrate, 30 mM EDTA, 0.15 M NaCl, and pH 6.0) for 10 minutes at 37°C with mechanical agitation. For all experiments media was changed twice weekly with timepoints at three, seven or 14 days as appropriate. All experiments were conducted on three biological replicates (*N* = 3), with each biological replicate cultured in technical triplicate at each timepoint and variable for each method of analysis (*n* = 9).

### 2.4 Modification of culture conditions

Culture surfaces were modified though overnight incubation on a shaker at room temperature with 500 μL per well of 2% (v/v) gelatin (Sigma‐Aldrich), 50 μg/mL fibronectin (Sigma‐Aldrich) or 20 μg/mL Laminin‐521 (Appleton Woods, Birmingham, UK) in PBS. Wells were then washed with 1 mL PBS before seeding.

Media composition was modified through use of either DMEM (10% v/v FBS, 200 units/mL penicillin, 200 μg/mL streptomycin, 0.5 μg/mL amphotericin, 100 mM sodium pyruvate, and 10 μM Ascorbic acid‐2‐phoshate) or αMEM (10% v/v FBS, 1× v/v Glutamax [Invitrogen Life Technologies, Falls under thermo fisher scientific], 200 units/mL penicillin, 200 μg/mL streptomycin, 0.5 μg/mL amphotericin, and 10 μM ascorbic acid‐2‐phosphate).

To test the influence on hypoxia, NC cells were cultured in 2% O_2_, 5% CO_2_ and 93% N_2_ or 20% O_2_, 5% CO_2_ and 75% N_2_ for 14 days as appropriate in αMEM media on laminin‐521‐coated plates. Media was degassed prior to use and all media changes and assays were conducted under hypoxic conditions.

To test the influence of osmolarity, NC cells were cultured in 300 mOsm/L αMEM media (10% v/v FBS, 1× Glutamax, 200 units/mL penicillin, 200 μg/mL streptomycin, 0.50 μg/mL amphotericin, and 10 μM Ascorbic acid‐2‐phosphate) or 400 mOsm/L αMEM media (10% v/v FBS, 1X v/v Glutamax, 200 units/mL penicillin, 200 μg/mL streptomycin, 0.50 μg/mL amphotericin, 10 μM ascorbic acid‐2‐phosphate, 1% 5 M NaCl, and 1% 0.4 M KCl)^36^ as appropriate in 2% O_2_, 5% CO_2_ and 93% N_2_, 37°C with laminin‐521‐coated surfaces.

Finally, to assess the influence of substrate stiffness, NC cells were cultured on Softwell Plates containing easy coat gels at 0.5 and 4 kPa or no gel (Cell Guidance Systems, Cambridge, UK), coated with laminin‐521 prior to culture with 400 mOsm/L αMEM media in 2% O_2_, 5% CO_2_ and 93% N_2_, 37°C.

### 2.5 Assessment of NC cell viability and morphology

Cells were incubated with 1 mL of 5% Alamarblue in appropriate media at day 3, 7, and 14 timepoints. Plates were incubated at 37°C for 3 hours. Following incubation, 100 μL of 5% Alamarblue in media was removed and read using a BioTek FLx800 at wavelengths 540/35 (ex.) and 590/20 (em.), sensitivity 50.

For lactate dehydrogenase (LDH) assay, media containing non‐adherent cells was removed at day three, and adherent NC cells were detached using 1× Trypsin‐EDTA for 5 minutes at day three, seven and 14 timepoints. Both populations were lysed using 2% Triton X‐100/HBSS for 1 hour at 37°C in the dark and 100 μL of each solution was transferred to a 96‐well plate and combined with 100 μL of reaction mixture (prepared as described in Roche Cytotoxicity Detection Kit). Plates were incubated for 30 minutes in the dark at room temperature and then read at 490 nm by Thermo Multiskan FC.

At 3, 7, and 14 days, media was removed from live cells and replaced with PBS. These cells were then imaged using an Olympus/MMI microscope. Estimation of percentage coverage of NC and NP‐like morphologies was achieved by eye based on presence/absence of vacuolar structures through division of three low‐magnification images into sectors.

### 2.6 Gene expression analysis

Porcine NP and AF tissue, dissected as described above, was snap frozen in liquid nitrogen and homogenized using a Retsch MM301 tissue homogenizer for 3 minutes, then RNA extracted using TRIzol according to manufacturer's recommendations. RNA was also extracted from cultured NC cells using TRIzol and, in all cases, RNA was reverse transcribed to cDNA using a High‐Capacity cDNA Reverse Transcription Kit (Applied Biosystems, Fisher Scientific UK Ltd, Loughborough). Quantitative real‐time PCR (qPCR) was performed using the SYBR green method for MRPL19, CD24, KRT8, KRT18, KRT19, and T (Table 1) on a StepOnePlus Real‐Time PCR System following manufacturer guidelines and 10 ng cDNA. Data were normalized to the pre‐validated reference gene MRPL19 and expression of NC marker genes presented as 2^−ΔCt^ using Graphad Prism 7.0.

**Table 1:** Sequences for qPCR primers

Gene name	Forward primer sequence	Reverse primer sequence
MRPL19	CTATTGAAGGACAAGGAGTT	GGCATCTCGCAAGTATAG
CD24	CTTTGGGTTTGACATTGT	GAATCTAGCACTCTTATTGAA
KRT8	CCTCTGATGTCCTGTC	TGAATTGGCTTGGAGT
KRT18	CAGGGACTGGAGTCATTA	GCATTGTCCACAGAACTT
KRT19	AAGAAGAACCACGAGGAG	GGAGCCGAATCAACCT
T (Brachyury)	CCTTCAGCAAAGTCAAGC	CGTACTTATGTAAGGAGTTCAG

All primers used at a final concentration of 500 nM.

## 3 RESULTS

### 3.1 Comparison of human and porcine NC morphology and phenotype

**Figure 1:**
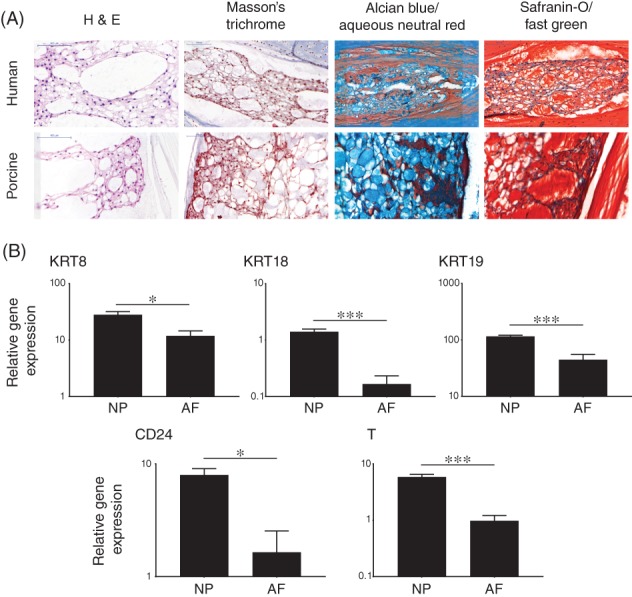
Validation of porcine nucleus pulposus as an appropriate model of human NC cells. (A): Histology of native foetal IVD tissue and young porcine NP tissue. Human foetal NP tissue (18‐weeks post‐conception) and young porcine NP tissue (6‐weeks post‐natal) were stained using hematoxylin and eosin (H&E), Masson's trichrome, alcian blue/aqueous neutral red and safranin O/fast green. (B): Expression of selected NC phenotypic marker genes (KRT8, KRT18, KRT19, CD24, and T) in porcine NP and AF (*N* = 3). Values shown represent mean 2^−ΔCt^ (±SE). *P* < 0.05 (*), *P* < 0.01(**), *P* < 0.001(***)

Histological analysis revealed similarities between 18‐week post‐conception human and 6‐week post‐natal porcine IVD tissue (Figure 1A). Hematoxylin and eosin (H&E) staining demonstrated the comparable morphology of foetal human NP tissue and porcine NP tissue with presence of large, vacuolated notochordal cells clearly visible and consistent between samples. Masson trichrome clearly showed the presence of abundant keratins (red) in both human and porcine NP tissue. Alcian blue staining demonstrated a high level of glycoprotein deposition in the NP region of both species. Similarly, Safranin‐O staining showed a high amount of glycosaminoglycans (GAGs) in the NP regions; glycoproteins and GAGs were also visible in surrounding tissue.

qPCR analysis demonstrated significantly higher expression of a panel of previously‐identified NC markers (KRT8, KRT18, KRT19, CD24, and T) in porcine NC cells compared to AF cells (Figure 1B). This paralleled the expression patterns of these genes in the human system as previously described.^52^, ^53^

### 3.2 Porcine NC cell attachment, morphology, and gene expression at day three

**Figure 2:**
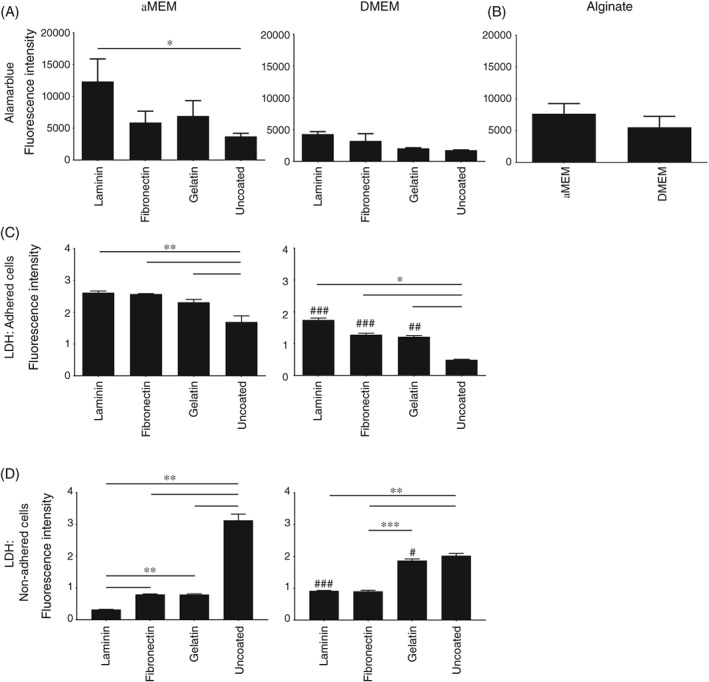
Viability and adherence of porcine NC cells in culture at day three. Comparison of Alamarblue activity and in two‐dimensional (2D) culture on (A) coated or uncoated surfaces or in (B) alginate beads in αMEM and DMEM. C: LDH activity in non‐adhered and adhered populations in 2D culture on coated or uncoated surfaces in αMEM and DMEM. Values shown represent mean absorbance (±SE). * Indicates significance between alternative surface coatings in the same media. # Indicates significance on the same surface coating in alternative media. *N* = 3; *n* = 9 for all experiments. *P* < 0.05 (* or #), *P* < 0.01 (** or ##), *P* < 0.001 (*** or ###)

Establishment of an initial adherence, morphology and gene expression profile at an early timepoint in culture set an important baseline for measuring proliferation and phenotypic changes over time. LDH data for adhered and non‐adhered porcine NC cells and Alamarblue for adhered porcine NC cells at day three indicated that surface coatings and media choice had significant impacts upon attachment (Figure 2). In particular, a significantly greater Alamarblue activity, and therefore number of attached cells, was observed in porcine NC cultures on laminin‐coated surfaces compared to uncoated surfaces (Figure 2A). A baseline for alginate beads was established using Alamarblue but did not show significant differences in population size between media choice (Figure 2B). Similarly, LDH data (Figure 2C) indicated a significantly greater number of attached porcine NC cells on coated surfaces compared to those on uncoated surfaces with both αMEM and DMEM. Examination of non‐adherent populations by LDH assay revealed complimentary results; cultures on uncoated surfaces showed significantly greater non‐adhered populations than coated surfaces and a greater number of non‐adherent cells were present in cultures on laminin and gelatin in DMEM compared with those in αMEM. This clearly defined the initial value of surface coatings and media choice. Crucially, use of αMEM media and laminin‐coated surfaces were shown to display the greatest cell attachment.

**Figure 3:**
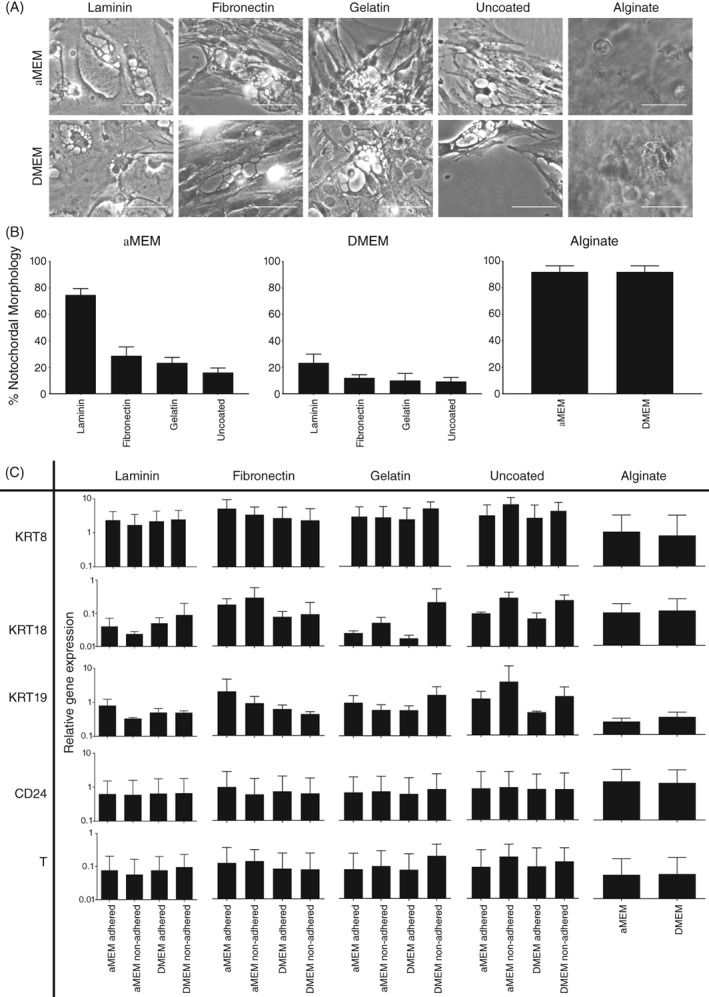
Morphology and phenotype of porcine NC cells in culture at day three. (A): Morphology of porcine NC cells in culture in αMEM or DMEM media on coated surfaces and in alginate at day three. Representative images of morphology and proportion of cells with NC‐like morphologies are shown for cultures on laminin, fibronectin, gelatin‐coated and uncoated tissue culture‐treated polystyrene and in alginate beads. (B): The mean proportion of cells with NC‐like morphologies from three wells (±SE) are represented graphically. Scale bars = 100 μm. (C): Gene expression of KRT8, KRT18, KRT19, CD24, and T at day 3 of culture on coated or uncoated tissue culture surfaces and in alginate beads. Each two‐dimensional (2D) culture graph represents non‐adhered and adhered populations in αMEM and DMEM media. Values shown represent mean 2^−ΔCt^ (±SE). No significant differences were observed. *N* = 3; *n* = 9 for all experiments

Media choice and use of surface coatings had a significant impact on morphology and gene expression profile at day three (Figure 3). Porcine NC cells in alginate culture displayed a classical NC morphology irrespective of media choice (Figure 3A). The highest percentage NC morphology (~70%) with clearly visible vacuoles was observed in porcine NC cultures in αMEM media on laminin‐coated surfaces, with cultures on fibronectin, gelatin and uncoated surfaces showing much lower percentage NC morphology (Figure 3B). Cultures on 2D surfaces in DMEM demonstrated a similar pattern compared to those in αMEM, but the percentages of cells with NC morphology were lower throughout. The NC‐like morphologies on all 2D surfaces showed a less rounded morphology than those in alginate cultures. However, these were still defined as NC‐like due to the clearly visible vacuole structures.

qPCR analysis of NC marker gene expression (Figure 3C) demonstrated no significant differences between cultures in αMEM and DMEM, between NC cell cultures in alginate and on any of the 2D surfaces, or between adherent and non‐adherent populations. This showed that gene expression is comparable between our 2D coated surfaces and current gold standard, alginate. Overall, laminin‐coated surfaces and in αMEM media were preferred conditions as the greatest cell adherence, the most NC‐like morphology of 2D cultures and a gene expression of selected NC markers comparable to alginate were observed in cultures under these conditions.

### 3.3 Porcine NC cell proliferation, morphology, and gene expression over 14 days

**Figure 4:**
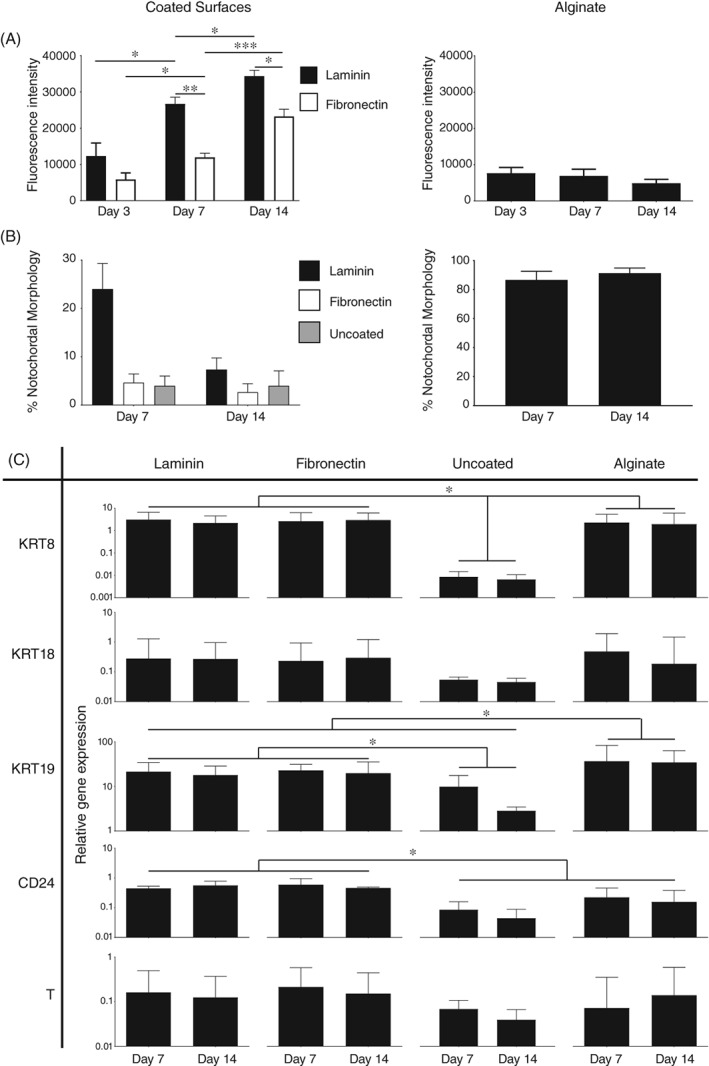
Proliferation and phenotype of porcine NC cells over a 14‐day culture period. (A): Alamarblue activity of adherent porcine NC cells following 3, 7 and 14 days in culture (±SE) in αMEM on laminin or fibronectin‐coated surfaces or in alginate beads. *P* < 0.05 (*), *P* < 0.01(**), *P* < 0.001(***). (B): Proportion of porcine NC cells with NC‐like morphology at day seven and 14 in αMEM media on laminin or fibronectin‐coated surfaces or uncoated tissue culture‐treated polystyrene (TCP), or in alginate beads. Graphical values represent the mean proportion of cells with NC‐like morphologies from three wells (±SE). (C): Gene expression of KRT8, KRT18, KRT19, CD24, and T at day 7 and 14 of culture in αMEM media on laminin or fibronectin‐coated surfaces or uncoated TCP, or in alginate beads. Values shown represent mean 2^−ΔCt^ (±SE). *P* < 0.05 (*). *N* = 3; *n* = 9 for all experiments

Significant differences in proliferation and gene expression were observed more clearly over the 14‐day culture period (Figure 4). Porcine NC cells in alginate culture did not display an increase in cell number over the 14‐day culture period, but a significant increase in population size was apparent on both laminin and fibronectin‐coated surfaces. At day seven and 14, significantly greater cell numbers were observed in NC cultures on laminin‐coated surfaces compared to fibronectin‐coated surfaces (Figure 4A).

At both days seven and 14 there was retention of a proportion of cells displaying NC‐like morphologies in alginate culture; however, retention of NC‐like morphology was reduced on 2D surfaces (Figure 4B). While cultures on laminin‐coated surfaces retained over 20% NC‐like morphology at day seven, this had decreased to below 10% by day 14. Cultures on fibronectin or uncoated surfaces retained fewer than 5% NC‐like morphologies by day seven and remained low at day 14. Importantly, significantly greater retention of NC gene expression was apparent on coated surfaces and in alginate compared to cultures on uncoated surfaces (Figure 4C). This defined the value of coated surfaces compared to uncoated and showed that porcine NC cells cultured on 2D surfaces demonstrated comparable gene expression to alginate culture. As such laminin‐coated surfaces provided the greatest proliferation, retention of morphology of the 2D surfaces and retention of gene expression, which indicated these as the most suitable conditions.

### 3.4 Proliferation, morphology, and gene expression under conditions mimicking the in vivo NP environment

**Figure 5:**
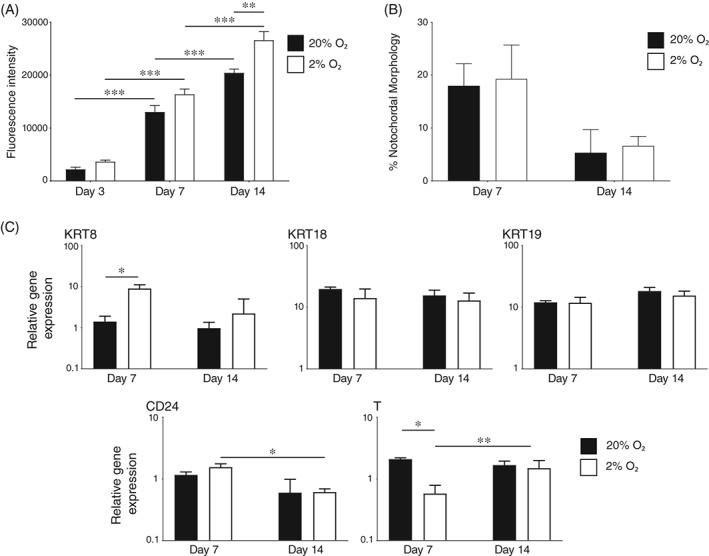
Effect of hypoxia on proliferation and phenotype of porcine NC cells. (A): Alamarblue activity of adherent porcine NC cells following 7 and 14 days in culture (±SE) in αMEM media on laminin‐coated surfaces in 20% or 2% O_2._ (B): Proportion of cells with NC‐like morphology at day 7 and 14 in αMEM media on laminin‐coated surfaces in 20% or 2% O_2_. Graphical values represent the mean of three wells (±SE). (C): Gene expression of KRT8, KRT18, KRT19, CD24 and T at day 7 and 14 of culture in αMEM media on laminin‐coated surfaces in 20% or 2% O_2_. Values shown represent mean 2^−ΔCt^ (±SE). *P* < 0.05 (*), *P* < 0.01 (**), *P* < 0.001 (***). *N* = 3; *n* = 9 for all experiments

Porcine NC populations cultured in 2% O_2_ were significantly greater at day 14 compared to those in 20% O_2_ (Figure 5A). The percentage of cells with NC‐like morphologies were similar in both cultures under 2% O_2_ and 20% O_2_ (Figure 5B) and the observed loss of these morphologies occurred in a similar pattern to earlier data (Figure 4B). Significant differences in expression of selected NC genes were not observed at day 14, although expression of KRT8 was greater and T lower in 2% O_2_ compared to 20% O_2_ at day seven (Figure 5C). Hypoxic conditions were considered preferable due to the increase in proliferation and no detriment to phenotype.

**Figure 6:**
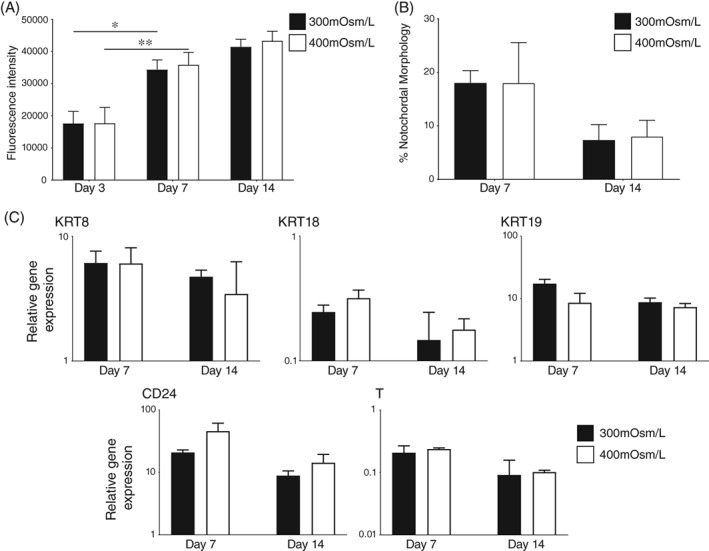
Effect of osmolarity on proliferation and phenotype of porcine NC cells. (A): Alamarblue activity of adherent porcine NC cells following 7 and 14 days in culture (±SE) in 2% O_2_ on laminin‐coated surfaces in 300 mOsm/L or 400 mOsm/L αMEM media. (B): Proportion of cells with NC‐like morphology at day 7 and 14 in 2% O_2_ on laminin‐coated surfaces in 300 mOsm/L or 400 mOsm/L αMEM media. Graphical values represent the mean of three wells (±SE). (C): Gene expression of KRT8, KRT18, KRT19, CD24, and T at day 7 and 14 of culture in 2% O_2_ on laminin‐coated surfaces in 300 mOsm/L or 400 mOsm/L αMEM media. Values shown represent mean 2^−ΔCt^ (±SE). *P* < 0.05 (*), *P* < 0.01 (**). *N* = 3; *n* = 9 for all experiments

No significant differences were observed in cell number (Figure 6A), retention of NC‐like morphologies (Figure 6B) or expression of selected genes (Figure 6C) in 300 mOsm/L αMEM media compared to 400 mOsm/L αMEM media. However, while no improvement was evident, there was no detrimental effect with changes in osmolarity.

**Figure 7:**
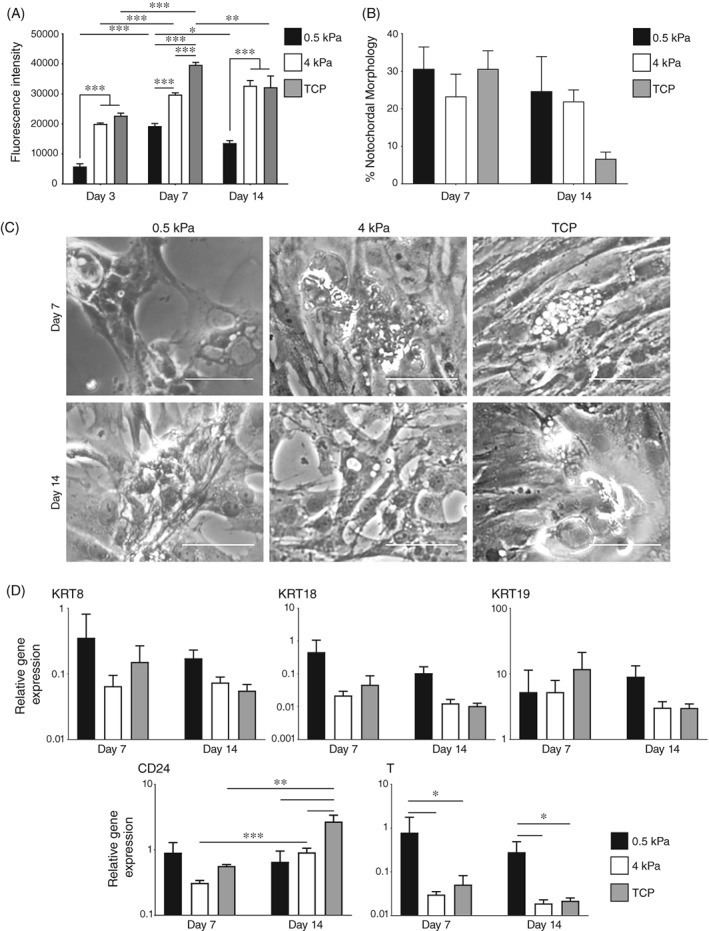
Effect of substrate stiffness on proliferation and phenotype of porcine NC cells. (A): Alamarblue activity of adherent cells following 7 and 14 days in culture (±SE) on laminin‐coated surfaces at 0.5 and 4 kPa or on laminin‐coated tissue culture‐treated polystyrene (TCP) in 400 mOsm/L αMEM media, under 2% O_2_. *P* < 0.05 (*), *P* < 0.01(**), *P* < 0.001(***). (B): Proportion of cells with NC‐like morphology in culture on laminin‐coated surfaces at 0.5 and 4 kPa or on coated TCP in 400 mOsm/L αMEM media, under 2% O_2_. Proportion of cells with NC‐like morphologies is shown for day 7 and 14. Values represent the mean proportion of cells with NC‐like morphologies from three wells (±SE). (C): Morphology of porcine NC cells in culture on laminin‐coated surfaces at 0.5 and 4 kPa or on coated TCP in 400 mOsm/L αMEM media, under 2% O_2_. Representative images of morphology and proportion of cells with NC‐like morphologies are shown for day 7 and 14. Scale bars = 100 μm (D): Gene expression of KRT8, KRT18, KRT19, CD24, and T at day 7 and 14 of culture in on laminin‐coated surfaces at 0.5 and 4 kPa or on coated TCP in 400 mOsm/L αMEM media, under 2% O_2_. Values shown represent mean 2^−ΔCt^ (±SE). *P* < 0.05 (*), *P* < 0.01 (**), *P* < 0.001 (***). *N* = 3; *n* = 9 for all experiments

Alterations to surface stiffness demonstrated greater impacts on proliferation and phenotype (Figure 7). Significantly lower cell numbers were observed on 0.5 kPa surfaces at each timepoint compared to stiffer surfaces. Cultures grown on laminin‐coated 4 kPa surfaces showed similar attachment and proliferation to cultures on laminin‐coated TCP (Figure 7A). Similar proportions of NC‐like morphologies were observed at day seven between cultures on surfaces at 0.5 and 4 kPa and on laminin‐coated TCP (Figure 7B,C). However, cultures on 0.5 kPa surfaces retained a much higher proportion of cells with NC‐like morphologies (~25%) at day 14, as did 4 kPa surfaces (~20%) compared to the patterns observed in cultures on laminin‐coated TCP (both here and in earlier data). It should also be noted that these NC‐like morphologies were slightly different in appearance based upon the surface stiffness, with morphologies on 0.5 kPa surfaces being more similar to those observed previously described in alginate. There were no significant differences present in the expression of KRT8, KRT18 or KRT19 but a significantly greater expression of T was observed in cultures on 0.5 kPa surfaces (Figure 7D).

## 4 DISCUSSION

NC cells have been shown to possess a number of therapeutically valuable qualities which make them a potentially ideal cell source for cell‐based therapies for DIVD and low‐back pain;^4^, ^5^, ^6^, ^7^, ^24^, ^25^, ^26^, ^28^, ^29^, ^30^, ^31^, ^54^ however, to date, a method of culture that allows for proliferation while retaining phenotype has not been described. We have developed a NC cell culture system that retains morphology and expression of known NC marker genes in line with the current gold standard, alginate, while also promoting cell proliferation. The first stage in development of this system was to define the suitability of porcine NC as an appropriate model for human NC cells. Known NC markers identified in human NC cells (KRT8, KRT18, KRT19, CD24, and T)^52^, ^53^ were found to be expressed in directly extracted porcine NC cells and histological staining demonstrated similarities in morphology and glycoprotein, keratin and GAG content of the extracellular matrix in both human and porcine tissue.

The first difficulty to overcome with design of a NC culture system in monolayer is cell adherence. It has previously been described that NC cells will not adhere to TCP until day six, with only cells of an adult NP‐like cell morphology adhering prior to this.^55^ Results here demonstrated high proportions of adherence on coated surfaces. In addition, there was no significant difference in gene expression between non‐adhered and adhered populations at day 3. Reports within the literature indicate that porcine NP is 80% to 88% NC,^37^, ^51^ therefore, our observed proportion of adhered: non‐adhered cells strongly suggests that our adhered populations are significantly NC.

The greatest adherence was observed in αMEM media and on laminin‐521 coated surfaces. These findings are in line with work from Rastogi et al, that indicated greater adherence of rat NC cells in αMEM media.^38^ There are many differences between DMEM and αMEM compositions; in this case, the most prominent of these was glucose concentration, with αMEM having a lower glucose composition than DMEM. High‐glucose levels, such as those in DMEM media, have been linked to rat NC cell senescence and apoptosis,^39^, ^56^ while low‐glucose levels have been suggested to promote rat NC cells viability and proliferation.^57^ The observed increased adherence on coated surfaces are likely due to the presence of integrins (immature porcine—*α*6, *β*1, and *β*4) (mature human NP *α*1, α3, *α*5, *α*6, and *β*1)^37^, ^41^, ^51^, ^58^, ^59^ and their interactions with extracellular matrix components, most prominently laminin.^41^, ^51^, ^58^ These findings are consistent with results from Gilchrist et al, which suggested that coated surfaces, particularly laminin isoforms 332 and 511, may promote greater adherence of porcine NC cells.^37^ Expansion of the culture period to 14 days demonstrated proliferation of NC cells on coated surfaces with the greatest number of cells being observed on laminin‐ coated surfaces. In addition, the major limitation with alginate bead culture was highlighted as significant changes in cell number were not observed over the entire culture duration, as expected.^32^ Crucially, NC cell marker gene expression was retained over 14 days on laminin and fibronectin‐coated surfaces, but not on uncoated surfaces highlighting the importance of such coating on maintenance of phenotype.

The addition of 2% O_2_ to mimic the young IVD^60^ resulted in a significant increase in NC cell proliferation by day 14. This increase in proliferation may be due to the action of HIF‐1α. Commonly, under normoxia, HIF‐1α is marked for degradation by prolie‐hydroxylase‐2 and von‐hippel‐lindau ubiquitin ligase complexes and is deactivated by factor inhibiting HIF‐1 protein. Under hypoxia HIF‐1α is stable as these factors are downregulated.^61^, ^62^, ^63^ Unusually, in NC cells HIF‐1α remains stable under normoxic conditions.^64^ However, a significant increase in HIF‐1α transcriptional activity has been detected in 2% O_2_ vs 20% O_2._ 65 While this does not influence GAPDH, GLUT‐1, and GLUT‐3,^65^ HIF‐1α has been shown to interact with a wide range of proliferation related factors including CD73, CTGF, ENG, IGFBP3, ITF, MET, NR4A1, REDD1, RORα4, STK15, TERT, TGFβ3, and WT1.^66^ Therefore, it could be suggested that the elevated HIF‐1a has effects in promotion of proliferation and the inclusion of hypoxic conditions help realize our aims. Importantly, porcine NC cells remained proliferative with the addition of hyperosmolar media and lower stiffness surfaces mimicking juvenile NP or adult NP.^37^, ^42^

Culture on coated surfaces did not display retention of NC‐like morphology as effectively as alginate culture, although cultures on laminin‐coated surface did display high levels of NC‐like morphology early in the culture period. The addition of 2% O_2_ or hyperosmolar media did not result in significant effects on morphological retention. If it is assumed that vacuoles exist as a protective measure against hypoosmotic stresses induced by mechanical and loading stresses,^46^ it would be reasonable to expect an improvement in morphological retention with hyperosmolar media as had previously been described by Spillekom et al, using a canine model in 3D culture.^36^ This loss of morphology was most likely due to the influence of the TCP underlying the surface coating. There is precedent for cells to respond to the stiffness of an underlying culture surface.^67^ Therefore, it is likely that the high stiffness of TCP is more prominent than the influence of the surface coating or hyperosmolarity with reference to morphology. This is reinforced by the improved retention of NC morphologies observed at day seven through to day 14 of culture on the more in vivo‐like 0.5 and 4 kPa surfaces compared to TCP, highlighting the importance of substrate stiffness in retention of morphology.

It is particularly interesting that while morphology was lost rapidly in 2D culture, gene expression profiles could be maintained during expansion. Crucially, the data demonstrate the importance of surface coating on retention of phenotype, as while laminin and fibronectin‐coated surfaces maintained phenotype compared to culture in alginate beads, expression of NC markers by cells on uncoated surfaces was significantly downregulated by day 14. As such, culture on laminin or fibronectin‐coated surfaces is able to maintain gene expression at a comparable level to alginate bead culture with the added value of proliferation.

Modification of microenvironmental parameters did not result in substantial changes in phenotype, with the most profound effect being an increase in T expression by NC cells on 0.5 kPa laminin‐coated substrates compared to laminin‐coated TCP. Previous studies have suggested an improvement in expression of KRT8 in porcine NC^34^ and an increase in T and KRT18 in canine NC cells^36^ under hypoxic and hyperosmolar conditions respectively. However, both studies were conducted under 3D conditions in alginate, rather than in monolayer. It is clear that surface stiffness is a crucial element in the development of a monolayer culture system for NC cells. The profound effects of lower stiffness surfaces may link to cell spreading. Work from numerous groups has indicated that cell spreading is heavily regulated by culture or matrix stiffness and that this may have significant effects both proliferation^68^, ^69^ and cell phenotype.^70^, ^71^ Gilchrist et al, have previously shown a similar pattern using porcine‐derived NC cells. It was observed that culture on a softer surface lead to reduced cell spreading and the formation of cell clusters.^43^ In addition, it was found that cultures on softer surfaces promoted greater GAG expression than those on stiffer substrates. Similarly to this, our results would indicate that porcine NC cells retain a more NC‐like morphology and gene expression profile on softer substrates. This is likely due to the adaptation of these cells to their in vivo environment and the particularly soft nature of this tissue in comparison to TCP (ie, 25 000‐200 000 times softer).^37^, ^42^

## 5 CONCLUSION

Overall, this study defines an optimized system for culturing NC cells, incorporating laminin‐521‐coated surfaces and αMEM media adjusted to 400 mOsm/L and 2% O_2_. Substrate stiffness is also an essential consideration, with soft substrates promoting retention of phenotype. Application of such a system will allow for expansion of NC cells with retention of phenotype, hence enabling further phenotypic and functional studies. Adoption of a standardized culture system for expansion of NC cells will enable more robust comparisons between experimental studies from different laboratories to be made. Furthermore, similarities between porcine and human NC cells demonstrated here suggest this system may be appropriate for future transfer to human NC cells to study their regenerative potential in more detail.
